# Update on Imaging of Ovarian Cancer

**DOI:** 10.1007/s40134-016-0157-9

**Published:** 2016-04-09

**Authors:** Rosemarie Forstner, Matthias Meissnitzer, Teresa Margarida Cunha

**Affiliations:** Department of Radiology, Landeskliniken Salzburg, Paracelsus Medical University, Müllner Hauptstr. 48, 5020 Salzburg, Austria; Serviço de Radiologia, Instituto Português de Oncologia de Lisboa Francisco Gentil, Rua Prof. Lima Basto, 1099-023 Lisbon, Portugal

**Keywords:** Ovarian neoplasm/diagnosis, Neoplasm staging, Ovarian neoplasm/therapy, Diagnostic imaging, Computed tomography, Diagnostic imaging

## Abstract

This review will make familiar with new concepts in ovarian cancer and their impact on radiological practice. Disseminated peritoneal spread and ascites are typical of the most common (70–80 %) cancer type, high-grade serous ovarian cancer. Other cancer subtypes differ in origin, precursors, and imaging features. Expert sonography allows excellent risk assessment in adnexal masses. Owing to its high specificity, complementary MRI improves characterization of indeterminate lesions. Major changes in the new FIGO staging classification include fusion of fallopian tube and primary ovarian cancer and the subcategory stage IIIA1 for retroperitoneal lymph node metastases only. Inguinal lymph nodes, cardiophrenic lymph nodes, and umbilical metastases are classified as distant metastases (stage IVB). In multidisciplinary conferences (MDC), CT has been used to predict the success of cytoreductive surgery. Resectability criteria have to be specified and agreed on in MDC. Limitations in detection of metastases may be overcome using advanced MRI techniques.

## Introduction

From a clinical perspective, ovarian cancer remains a major challenge. Despite advances in medicine over the past decades, only minor improvement in 5-year overall survival has been achieved in patients diagnosed with advanced epithelial ovarian cancer [1]. This cancer is the most lethal among the pelvic cancers, and cancer-associated mortality is as high as for cervical cancer and uterine cancer combined [2]. One of the reasons is its late diagnosis with more than 60 % of patients presenting already with metastatic spread beyond the pelvis. In this scenario, episodes of tumor recurrence will develop, followed by chemo resistance, and subsequently these patients will succumb to their disease [3•]. Increased understanding of the molecular biology of ovarian cancer opens new perspectives, and targeted therapies are emerging [4]. Moreover, gene abnormalities have been identified in different cancers subtypes, which will provide the basis for a personalized management in patients with ovarian cancer [3•].

This review will focus on the most common cancer type, epithelial ovarian cancer (EOC). The other primaries including germ cell and sex-cord stromal cell ovarian cancer are extremely rare (<5 %) and differ in many aspects, but share the same staging classification with EOC [5, 6]. An update of recent advances regarding EOC will be provided with special emphasis on their impact on clinical radiological practice.

## New Insights in Ovarian Cancer Biology

The concept of ovarian cancer as a single disease has been revised. Epithelial ovarian cancer is now understood as a subsumption of diverse cancer entities that vary significantly clinically as well as pathologically and on a molecular level [7–9]. It comprises the following main cancer subtypes:

- high-grade serous,
- low-grade serous,
- endometrioid,
- clear cell and
- mucinous ovarian cancer [6, 11].

Differentiation of these subtypes is pivotal with regards to several aspects, such as biomarkers, precursor lesions, clinical presentation at diagnosis, prognosis, and response to treatment [5–11] (Table 1). Furthermore, considerable heterogeneity even within the same epithelial ovarian cancer subtype has been identified on a macroscopic and molecular level. This has also been attributed to the dedifferentiation within different implants of the same primary and thus the problem of tumor recurrence after initial response to therapy [3, 12].

**Table 1:** Clinico-pathological and radiological characteristics of ovarian cancer subtypes

Carcinoma subtype	HG-serous	LG-serous	Mucinous	Endometrioid	Clear cell
Percentage (%)	70–80	<5	3	10	5–10
Gene mutations	TP53, BRCA1/2	BRAF; KRAS	KRAS	PTEN;CTNNB-1	KRAS, PTEN, PIK3CA
Precursor	STIC	Serous cystadenoma borderline tumor	Mucinous cystadenoma borderline tumor	Endometriosis	Endometriosis, clear cell adenofibroma
Tumor morphology	Cystic and solid; solid; irregular contour	Solid and cystic; papillary projections; Psammoma bodies;	Large, cystic or solid; smooth contour	Smooth contour; solid and cystic; solid nodule in endometrioma	Large, thick wall; cystic with mural nodules protruding into lumen
Uni- or bilateral	Bilateral	Bilateral	Unilateral	Unilateral	Rarely bilateral
Dissemination	Diffuse abdominal	Abdominal	Ovary	Pelvic	Pelvic
Platinum-based-chemotherapy response	High	Intermediate	Low	High	Low
Prognosis	Poor	Intermediate	Good	Good	Intermediate

Adapted from references [3, 5, 11, 13]

**Fig. 1:**
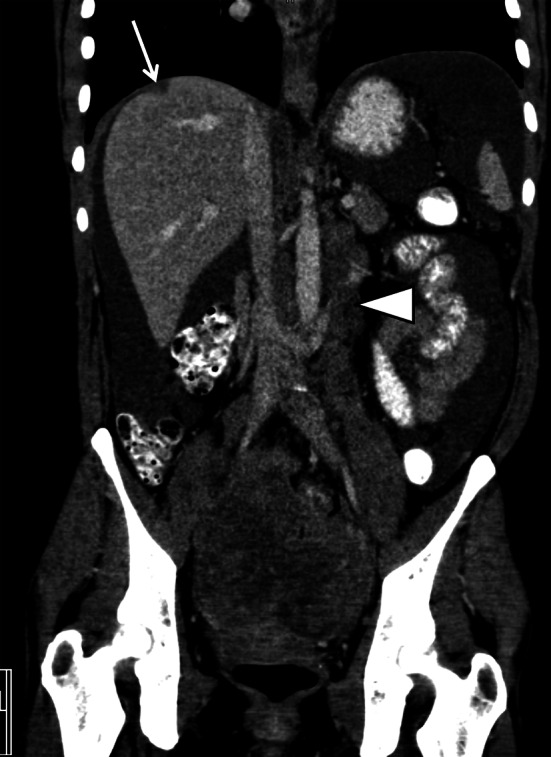
High-grade serous cancer FIGO stage IIIC with a bilateral solid adnexal mass, enlarged retroperitoneal lymph nodes (*arrowhead*), and large amounts of ascites and peritoneal implant (*arrow*) at the right diaphragm

From the radiological perspective, it is pivotal to understand that these subtypes of EOC may manifest with distinct radio-morphology of the primary ovarian mass and peritoneal metastatic traits at diagnosis may differ (Table 1) [7, 13]. The two serous cancer subtypes [high-grade (HGSC) and low-grade serous cancer (LGSC)] are fundamentally distinct neoplasms including molecular pathogenesis, their response to chemotherapy as well as prognosis [11]. Typical for HGSC is diffuse peritoneal dissemination at diagnosis, usually presenting with large amounts of ascites and peritoneal deposits throughout the abdominal cavity [13] (Fig. 1). Moreover, Vargas et al. reported an association between patterns of spread on CT imaging in different subtypes of HGSC based on the classification of ovarian cancer (CLOVAR) gene signatures. The mesenchymal CLOVAR subtype was found to be significantly more often associated with both diffuse mesenteric infiltration and peritoneal enhancement and adverse survival than the other genetic subtypes [14].

High-grade serous epithelial ovarian cancer is promoted by TP53 and BRCA1/2 mutations and seems to develop de novo and within only several months [13]. Conversely, low-grade serous ovarian cancer develops in a stepwise fashion from serous cystadenoma to serous Borderline cancer. In these tumors, KRAS and BRAF mutations are frequently identified, rather than BRCA1/2 mutations as in high-grade serous cancers [11]. The gradual malignant degeneration from a benign precursor lesion and slow growth is a feature that LGSC shares with the subtypes mucinous, endometrioid, and clear cell cancer. For the latter two cancer types, endometriosis has been identified as precursor [15].

High-grade serous ovarian cancer accounts for the vast majority (70–80 %) of ovarian cancers. There is increasing evidence that many of these cancers derive from tubal intraepithelial cells. This so called “serous tubal intraepithelial carcinoma” (STIC) theory proposes that high-grade serous ovarian cancer, tubal cancer, and primary peritoneal cancer share the common origin from serous tubal intraepithelial cancer [8, 9, 16, 17]. BRCA1 and BRCA2 mutation carriers have a 30–50 % life-time risk of developing ovarian cancer, mainly high-grade serous cancers [11]. Identification of these gene mutations, which occur in approximately 10 % of HGSCs, is important, as these patients have a better prognosis, and new therapeutic options can be offered [18].

## Prediction of Malignancy in Adnexal Masses

The clinical impact of defining whether an adnexal mass is benign or malignant is enormous. If a newly detected lesion carries a substantial risk of malignancy, treatment should be performed in a specialist oncology center [19]. Women believed to have advanced ovarian cancer usually require radical cytoreductive surgery followed by chemotherapy or alternatively neoadjuvant chemotherapy followed by interval debulking. Conversely, women with benign adnexal masses may be either treated conservatively or undergo simple resection by a general gynecologist [20]. Thus, predictive models have been developed to triage women presenting with adnexal masses to an appropriate treatment regimen [21•]. Findings of malignancy are listed in Table 2. For pre-surgical assessment of such an adnexal mass, transvaginal ultrasonography (US) combined with Doppler techniques is the first-line and best imaging technique. When a lesion is large or extends beyond the field of view of transvaginal US, complementary transabdominal US should be performed according to the 2013 American College of Radiology appropriateness criteria [22]. MRI is usually considered as complementary problem-solving modality [20]. Integration of additional clinical features (e.g., menopausal status) and serum biomarkers (CA-125) allow further risk stratification, e.g., as in the widely used risk of malignancy index (RMI).

**Table 2:** Imaging criteria for malignancy

Size	>4 cm
Morphology	Complex solid and cystic Solid enhancing component Thick septations >3 mm Papillary projections Central necrosis
Vascularization	Type 3 dynamic contrast curve
Additional findings	Ascites Lymph node enlargement Peritoneal carcinomatosis Organ invasion

Adapted from references [31, 40]

The value of gray scale and color Doppler US has been extensively analyzed by the International Ovarian Tumor Analysis (IOTA) group. The pattern recognition of specific ultrasound findings and assignment into categories of diagnostic certainty of malignancy is well established [23]. The IOTA reported a sensitivity of 91 % and specificity of 96 % of malignancy for a lesion in the categories highly and moderately confident to be malignant [23]. When performed by highly trained clinicians in women, this imaging technique was even equivalent to logistic regression models [24]. Various US-based approaches have been created and validated to optimize the pre-surgical diagnosis of adnexal tumors. These include scoring systems, rules models, logistic regression mathematical models, and the ADNEX model [21•, 25–28]. The IOTA “simple rules” assist in classifying adnexal masses as benign or malignant by assigning 5 US characteristics to each category [21•, 26]. These rules have been extensively studied and allow excellent prediction of malignancy (pooled sensitivity of 93 % and specificity of 95 %) [26]. In a meta-analysis from 2014 (analyzing 195 studies and 19 ultrasound risk models), the LR2 model and the simple rule**s** model yielded sensitivities of 88–95 and 89–95 %, respectively, and specificity of 77–88 and 76–85 %, respectively [21•, 27]. The authors concluded that these models are currently considered as best US-based models and strategies for use in clinical practice [21•]. The IOTA ADNEX model, a multiclass risk prediction model is designed to recognize specific histopathological entities [21•, 25]. A prospective multi-center study reported that the nine predictors of this ADNEX model not only allow differentiation of benign tumors from malignant tumors, but also enable categorization in five types of adnexal tumors (benign; borderline; stage I tumors; stage II-IV and metastatic tumors) [25]. However, it has to be mentioned that the exams were performed by experienced professionals, and there was not a central revision of pathology.

The ability to correctly characterize adnexal masses using subjective assessment directly correlates with the level of training/experience [21•, 24]. However, even with expert status 5–20 % of masses will remain indeterminate or difficult to classify [28]. These typically exhibit the following sonographic features: large size, uni- or multilocular with solid aspects, irregular walls and papillary projections, and multilocular cysts. The majority of these lesions are nevertheless benign tumors, mostly cystadenomas, cystadenofibromas, and fibromas [20]. In one study, 70 % of these lesions were benign and 16 % were invasive cancers and 14 % borderline malignant tumors [28].

**Fig. 2:**
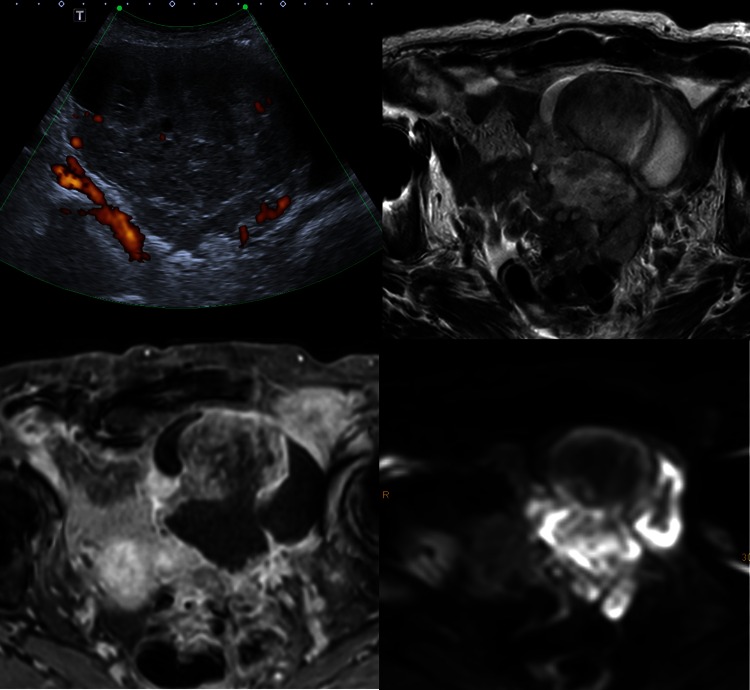
Ovarian cancer in an 87-year-old patient. Large inhomogeneous solid mass with cystic and necrotic areas and ascites in ultrasound (**a**) and T2W MRI (**b**, *arrows*). Typical findings supporting malignancy include highly vascularized solid areas (**c**) with restricted diffusion (**d**)

In the European Society of Urogenital Radiology (ESUR) guidelines, MRI is being recommended as a complementary tool in indeterminate US. An algorithmic approach using basic and problem-solving sequences will allow a confident diagnosis in the majority of cases and thus contribute to avoiding unnecessary surgery [20]. A systematic review confirmed the ability of MRI to confidently diagnose benign lesions in masses that were indeterminate at US using conventional techniques [29]. However, combining conventional MRI with the functional techniques diffusion-weighted imaging (DWI) and dynamic contrast enhancement (DCE) will further improve characterization of complex adnexal masses [30] (Fig. 2). DWI alone for differentiation between benign and malignant adnexal lesions is limited due to overlap of findings, although it will reliably exclude malignancy in cases where no high signal is identified within a solid mass on high b-value images [30, 31, 32•, 33, 34]. There is a reasonable body of evidence that ADC quantification is not useful for predicting benignity [33]. ADC entropy may have a potential for characterization of malignancy. In one study, analyzing 37 patients this technique showed significantly higher accuracy than visual and quantitative ADC assessment [35]. The technique of utmost importance for characterization of complex adnexal masses is semi-quantitative multiphase-dynamic contrast-enhanced MR [36, 37]. For this technique, it has been shown that factors related to tumor biological processes and neoangiogenesis are determinants in the contrast uptake dynamics in adnexal masses [38]. This technique is based on time-intensity curves acquired of solid aspects of adnexal lesions during multiphase-dynamic contrast-enhanced MRI. Comparison of the enhancement pattern with that of myometrium will allow to identify three types of enhancement curves which correlate with benign, borderline, and malignant lesions [39, 40•]. Type 1 time-intensity curves are characterized by a gradual uptake of contrast and has been more frequently encountered in benign than borderline and never in malignant lesions. Type 2 time-intensity curves, typical of borderline lesions, reflect an early uptake of gadolinium (though later than myometrium) followed by a plateau. Type 3 time-intensity curves are typical of malignant tumors, with an avid and early contrast uptake, followed by a wash-out [39, 40•]. This technique is also being used in the MRI ADNEX score system, a standardized MR imaging and reporting system for complex adnexal masses [40•]. The MRI ADNEX score divides masses into five categories from score 1 corresponding to no mass to score 5 that describes a probably malignant mass. A feasibility study demonstrated excellent reproducibility and interobserver agreement for various degrees of expertise [39]. Currently, a prospective multi-center validation study with more than 1600 patients is being conducted by the ESUR, with data expected to be published in 2016. Owing to false positive as well as false negative findings, currently, PET/CT is of no advantage over MRI for characterizing complex adnexal masses [41, 42].

## Revised FIGO Classification and Potential Impact on Radiological Interpretation

Ovarian cancer is surgically staged according to the FIGO or TNM staging classification. The FIGO system, which is most commonly used world-wide, has updated its classification, effective from 2014 on. Of note, this classification applies not only for EOC but also for sex-cord stromal and germ cell malignancies [5, 43•]. A revision of the classification seemed warranted in the light of new concepts in ovarian cancer biology, including immunohistochemical and molecular genetic analysis, overlap of histopathological features, new prognostic factors, differences in chemotherapy response, and the need for new treatment protocols [17, 43•, 44, 45].

In this new staging classification system, not only the tumor stage should be documented, but also the histological subtypes and grade.

Acknowledging the concept of a common origin of high-grade serosal tumors from tubal cells, the most important revision in the new staging classification includes that now ovarian, fallopian, and primary peritoneal cancer are seen as one entity. The other major change is the further subdivision of the stages III and IV. The rationale for subcategorization of stage IIIA1 is that according to evidence patients with isolated retroperitoneal lymph node metastases have a better prognosis than those who have extrapelvic peritoneal spread (now FIGO IIIB) [45, 46]. The findings in the various FIGO stages are seen in Table 3.

**Table 3:** FIGO classifications of ovarian cancer

FIGO stage	Subcategory and findings
I	A
	Tumor one ovary **or fallopian tube**
	B
	Both ovaries **or fallopian tubes**
	C
	One or both ovaries or fallopian tubes and
	C1: **surgical spill**
	C2: capsule ruptured or tumor on surface
	C3: malignant cells in ascites or peritoneal washings
**II**	A
	Extension/implants on uterus and/or ovaries and/or fallopian tubes
	B
	Extension to other pelvic intraperitoneal tissues
**III**	A
	A1 **Positive retroperitoneal lymph nodes (LN) only**
	A1(i): metastasis ≤10 mm
	A1(ii): metastasis >10 mm
	A2 **Microscopic extrapelvic peritoneal spread** **±** **LN**
	B
	Peritoneal implants outside pelvis up to 2 cm ± retroperitoneal LN
	C
	Peritoneal implants outside pelvis > 2 cm ± retroperitoneal LN; liver and/or spleen surface metastasis included
**IV**	A
	**Pleural effusion with positive cytology**
	B
	Parenchymal metastasis, metastasis to extraperitoneal organs, inguinal LN and LN outside abdominal cavity

Changes made to the version from 1998 are highlighted

Imaging findings in CT and MRI have also been adapted to the FIGO classification system [47, 48]. Thus, changes and key features relevant for pre-therapeutic imaging are highlighted in the following paragraph.

Of utmost importance is the understanding of merging of ovarian, fallopian tube, and primary peritoneal cancer in staging. This results in major ramifications for radiological reporting: fallopian tube and primary peritoneal cancers are no longer regarded as single entities and thus they are no longer staged differently than ovarian cancer [8, 9, 17]. In a malignant adnexal mass, differentiation of fallopian from ovarian origin has often been challenging. The classical findings of a sausage like adnexal mass or fallopian tube distension and a focal solid mass are only rarely visualized [49]. This needs no longer to be differentiated, which will facilitate radiological reporting. The other major change is that primary ovarian cancer no longer exists as a separate entity. The findings previously indicative of this disease, e.g., peritoneal metastases and normal ovaries constitute now a subtype of ovarian cancer. Stage I primary peritoneal cancer does not exist [17]. If it is limited to the pelvis, it has to be classified as stage II, but more often, it will present with stage III B or C with ascites and spread outside the pelvis at diagnosis.

The new staging classification also leads to the clarification of the lymph node status. Regional lymph nodes in ovarian cancer remain pelvic (internal and external iliac, obturator, and common iliac), presacral, and paraaortic and paracaval nodes. Conversely, inguinal lymph nodes are now considered as distant metastases (IVB). In contrast to other sites, smaller lymph node thresholds are used to assess CLNM (cardiophrenic lymph node metastases). A short axis cut-off of 5 mm has been suggested as normal cardiophrenic lymph nodes size [50]. A recent study reported 86 % positive predictive value for histologically proven CLNM using a short axis diameter of >7 mm in preoperative CT [51]. CLNM are associated with peritoneal dissemination and are found in approximately 30 % of advanced ovarian cancer [52]. These metastases occur typically in the anterior prepericardiac region, more commonly on the right than on the left side [51].

Stage IVA1 is characterized by pleural metastases proven either by positive cytology or biopsy. Stage IVB characterizes parenchymal metastases in the abdomen or extraabdominal lymph node metastases. Peritoneal implants on surface of liver and spleen (stage IIIC) have to be differentiated from parenchymal metastases in these organs. Umbilical metastases, Sister Mary Joseph nodes, are classified as metastases corresponding to stage IV B [5]. Transmural bowel invasion with mucosal involvement is also assigned to this category [5]. Conversely, rectum invasion defined as stage IIB, since it represents spread within the pelvis [5]. Quantification of ascites as small, moderate, or large should be included in the report, because it has shown to be related with survival [53].

## Prediction of Resectability in Ovarian Cancer

Comprehensive staging laparotomy and cytoreductive surgery followed by chemotherapy has been the standard of care in newly diagnosed advanced ovarian cancer [17]. There is evidence that cytoreduction is associated with increased survival in ovarian cancer. In the last years, the ultimate goal of cytoreduction has continuously been changed [17, 54]. Currently, a cut-off of 1 cm of residual tumor size is defined as optimal cytoreduction [55]. However, a trend toward ultraradical surgery with complete resection of all gross tumor deposits can be noted [55]. A recent meta-analysis of 18 studies with more than 13,000 patients proved positive impact of complete cytoreduction on median survival [56].

The best treatment for the advanced cancer stages IIIC and IV has been a subject of ongoing debate and much controversy over the last years [54, 57, 58]. Supporters of neoadjuvant chemotherapy refer to cancer biology and to the issue of high perioperative complications [54, 59]. Debulking rates differ significantly between different sites and countries. Optimal cytoreduction broadly ranges from 15 to 85 %, with high-volume oncologic centers attaining rates of 60–75 % [17, 60, 61]. To improve treatment stratification, particularly to select patients amenable or not for successful cytoreduction, various predictive tests have been published. Major determinants include clinical risk factors (e.g., age, performance status, obesity, and comorbidity), tumor markers (CA-125), and imaging, most commonly CT [33, 60, 61]. However, the problem in the preoperative assessment of resectability is that there is no general accepted model and that reproducibility is a major challenge due to different clinical practice. Other limitations include complexity of scoring systems and prediction models. Different resection rates are also attributed to subjective assessment of resectability based on surgeons experience and preference, on anesthesia support and different departmental policies [33].

**Fig. 3:**
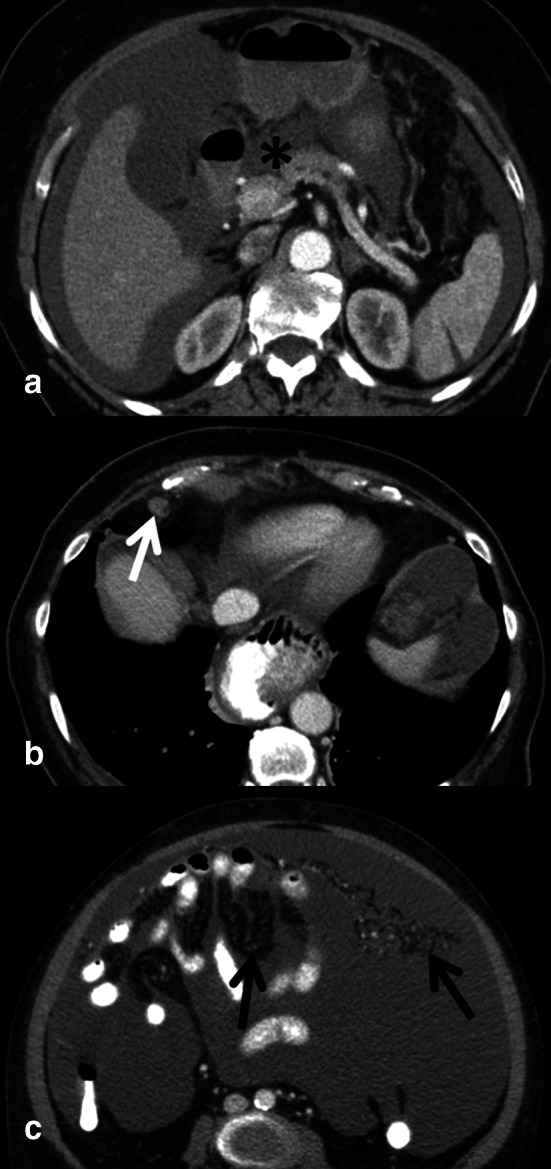
Subtle imaging findings indicative of advanced ovarian cancer spread: ascites in omental bursa (**a**, *asterisk*) lymph node (**b**, *arrow*) with a short axis diameter of >7 mm in the cardiophrenic fat above the diaphragm. In all ovarian cancer staging exams, mesentery and omentum should be scrutinized for band-like and reticular pattern (**c**, *arrows*) presenting peritoneal spread

**Fig. 4:**
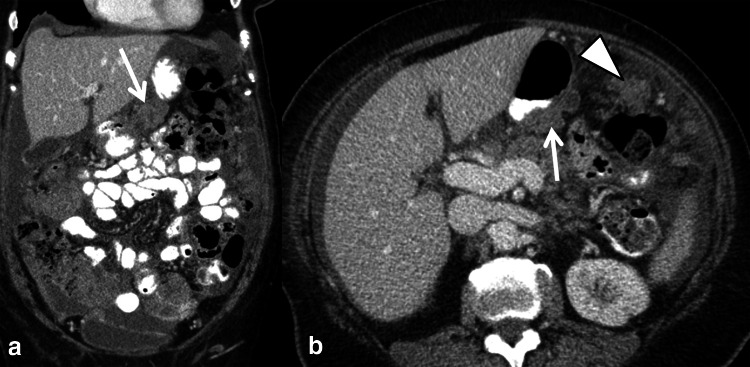
Excessive peritoneal metastases in the upper abdomen in high-grade serous cancer. Sites as in the omental bursa (*arrow*) and large deposits along the gastrocolic ligament (*arrowhead*) are findings indicative of non-optimal cytoreduction in most centers. These may synonymously also be termed as “difficult to resect”

Multidisciplinary consensus conferences (MDC) are the platform to define individualized optimal treatment regimen. If a patient with ovarian cancer will benefit from upfront surgery or rather from a neoadjuvant approach has to be discussed in the context of patient-related factors and surgical technical issues [33, 62•]. In this setting, the accurate mapping of tumor burden and distribution of disease by imaging plays a central role in treatment stratification and will thus also influence patient outcome [47, 48, 62•]. Site, size, and distribution of metastases have been used as radiological predictors for the outcome of cytoreductive surgery. CT images need to be scrutinized for subtle findings of peritoneal spread since these can change treatment decision (Fig. 3). Various CT criteria assessing different sites throughout the abdomen and CT scores without and with the incorporation of CA-125 or other clinical criteria have been proposed [33, 60, 61, 63, 64]. In the ESUR guidelines for staging ovarian cancer, large disease (>2 cm) in the upper abdomen around the liver and spleen, mesenteric deposits and lymph node metastases above the renal hilum were summarized as sites likely to be not optimally resectable [47] (Fig. 4). However, it was emphasized that resectability criteria may differ from center to center, and that predictive parameters have to be specified and agreed on in MDC [47]. Two recent publications of high-volume tumor centers addressed the value of CT for prediction of cytoreduction in ovarian cancer. A multi-center prospective trial of two major US cancer centers analyzed features to predict suboptimal cytoreduction. In 350 patients with surgically treated ovarian cancer, three clinical and six radiological criteria were significantly associated with suboptimal debulking [60]. Borley et al. analyzed radiological predictors associated with debulking success and requirement for bowel resection by logistic regression models. In their study, the presence of lung metastases >7 mm, pleural effusion, deposits >10 mm in size on large and small bowel mesentery, and infrarenal paraaortic lymph node metastases were associated with low success rate with debulking [61]. Despite advanced surgical techniques in both studies, bowel involvement was a major limitation for optimal cytoreduction. Thus, signs of bowel and mesenteric involvement have to be carefully analyzed in CT, like e.g., bowel wall thickening, adhesions, and mesenteric tethering [14, 48]. Since small size (<5 mm) peritoneal deposits are hard to see in CT, complementary laparoscopy may play a role in the preoperative assessment of ovarian cancer [57]. It seems that currently the role of PET/CT for primary ovarian cancer staging is limited, as it is not superior to CT alone, and treatment regimen is not changed [41]. There is a paucity of data of advanced MRI techniques for staging of ovarian cancer [33]. Low and Barone used the peritoneal cancer index based on MRI/DWI and DCE in 35 patients (5 with ovarian cancer) with peritoneal carcinomatosis. Radiological–surgical correlation yielded a high match of tumor sites [65]. Recently, these authors also reported the superiority of this advanced MRI technique compared to CT [66]. In a prospective comparative study with surgery as standard of reference whole-body MRI using DWI was superior to CT and to PET/CT in the challenging assessment of bowel serosal and mesenteric disease. Furthermore, metastases outside the abdomen could be detected similarly to PET/CT [67•]. Conversely, another comparative study found no significant differences between MRI, CT, and PET/CT for staging. However, PET/CT was more accurate for supradiaphragmatic metastases [68].

## Conclusion

Advances in immunohistochemistry and molecular genetics are the basis of new concepts in ovarian cancer. Imaging is integral in various aspects in assessing ovarian cancer. It is not only used as a diagnostic tool but is also a major determinant in triaging to personalized treatment. Sonography is an excellent modality to predict malignancy in adnexal masses and thus assists in reducing unnecessary surgeries.

The use of functional MRI techniques is further improving characterization of sonographically indeterminate masses of which the vast majority will be benign. The MRI ADNEX score provides standardized assessment and reporting of complex adnexal masses.

In MDC, imaging plays a central role for treatment stratification in ovarian cancer. It serves as a roadmap for surgery and is one of the major predictors for successful primary cytoreductive surgery. Currently, CT is the standard of care for staging patients with ovarian cancer. However, MRI using functional techniques is emerging as technique that may be able to overcome limitations of staging CT.

The revised FIGO classification has introduced some major changes radiologists have to be familiar with. This includes the fusion of fallopian tube and primary ovarian cancer, and new concepts regarding lymph node dissemination as well as distant metastases.

## References

[1] SEER cancer statistics review 1975–2012 seer.cancer.gov/statfacts/html/ovary.html. Accessed 19 Jan 2016.

[2] Cancer fact and figures 2015. American Cancer Society www.cancer.org. Accessed 19 Jan 2016.

[3] • Jayson GC, Kohn EC, Kitchener HC, Ledermann JA. Ovarian cancer. Lancet 2014; 384:1376–88. *This paper covers various aspects of ovarian cancer from genetics and molecular pathology to clinical management and the emerging role of targeted treatment.* 10.1016/S0140-6736(13)62146-7 24767708

[4] Schmid BC, Oehler MK (2014). New perspectives in ovarian cancer treatment. Maturitas.

[5] Höhn AK, Einenekel J, Wittekind C, Horn LC (2014). Neue FIGO-Klassifikation des Ovarial-Tuben und primären Peritonealkarzinoms. Pathologe.

[6] Kurman RJ, Carcangiu ML, Herrington CS, Young RH. Classification of tumours of the ovary. In: WHO classification of tumours, Vol. 6. 4th ed. Lyon: IARC Press; 2014.

[7] Lawrenson K, Gyther SA (2009). Ovarian cancer: a clinical challenge that needs some basic answers. PLOS Med..

[8] Kurman RJ, Shih IeM (2011). Molecular pathogenesis and extraovarian origin of epithelial ovarian cancer–shifting the paradigm. Hum Pathol.

[9] Kurman RJ, Shih IeM (2010). The origin and pathogenesis of epithelial ovarian cancer: a proposed unifying theory. Am J Surg Pathol.

[10] Banerjee S, Kaye SB (2013). New strategies in the treatment of ovarian cancer: current clinical perspectives and future potential. Clin Cancer Res.

[11] Rendi MH. Epithelial carcinoma of the ovary, fallopian tube, and peritoneum: histopathology. www.uptodate.com (2016). Accessed 19 Jan 2016.

[12] Sala E, Kataoka MY, Priest AN, Gill AB (2012). Advanced ovarian cancer: multiparametric MR imaging demonstrates response-and metastasis-specific effects. Radiology.

[13] Lalwani N, Prassad SR, Vikram R, Shanboghue AK (2011). Histologic, molecular, and cytogenetic features of ovarian cancers: implication for diagnosis and treatment. RadioGraphics.

[14] Vargas HA, Micco M, Hong SI, Goldmann DA (2015). Association between morphologic CT imaging traits and prognostically relevant signatures in women with high-grade serous ovarian cancer. A hypothesis-generating study. Radiology.

[15] Mandai M, Yamaguchi K, Matsumura N, Baba T, Konishi I (2009). Ovarian cancer in endometriosis: molecular biology, pathology, and clinical management. Int J Clin Oncol.

[16] Nik NN, Vang R, Shih IM, Kurman RJ (2014). Origin and pathogenesis of pelvic (ovarian, tubal, and primary peritoneal) serous carcinoma. Annu Rev Pathol.

[17] Mann JW, Chalas E, Valea FA. Cancer of the ovary, fallopian tube, and peritoneum: staging and initial management. www.UpToDate.com (2015). Accessed 19 Jan 2016.

[18] Bolton KL, Chenevix-Trench G, Goh C (2012). Association between BRCA1 and BRCA2 mutations and survival in women with invasive epithelial ovarian cancer. JAMA.

[19] Vernooij F, Heintz P, Witteveen E, van der Graaf Y (2007). The outcomes of ovarian cancer treatment are better when provided by gynecologic oncologists and in specialized hospitals: a systematic review. Gynecol Oncol.

[20] Spencer JA, Forstner R, Cunha TM, Kinkel K, On behalf of the ESUR Female Imaging Sub-Committee (2010). ESUR guidelines for MR imaging of the sonographically indeterminate adnexal mass: an algorithmic approach. Eur Radiol.

[21] • Kaijser J, Vandecaveye V, Deroose CM et al. Imaging techniques for the pre-surgical diagnosis of adnexal tumours. Best Pract Res Clin Obstet Gynaecol 2014; 28:683–95. State of the art r*eview of imaging in adnexal masses including US, MRI and PET/CT. Various models and studies of the IOTA group are outlined.* 10.1016/j.bpobgyn.2014.03.013 24780415

[22] www.acr.org/~/media/ACR/Documents/AppCriteria/Diagnostic/ClinicallySuspectedAdnexalMass.pdf.

[23] Valentin L, Ameye L, Savelli L (2011). Adnexal masses difficult to classify as benign or malignant using subjective assessment of gray-scale ad Doppler ultrasound findings. Logistic regression models do not help. Ultrasound Obstet Gynecol.

[24] Levine D, Asch E, Mehta TS, Broder J (2008). Assessment of factors that affect the quality of performance and interpretation of sonography of adnexal masses. J Ultrasound Med.

[25] Van Calster B, Van Hoorde K, Valentin L (2014). Evaluating the risk of ovarian cancer before surgery using the ADNEX model to differentiate between benign, borderline, early and advanced stage invasive, and secondary metastatic tumours: prospective multicentre diagnostic study. BMJ.

[26] Nunes N, Ambler G, Foo X (2014). Use of IOTA simple rules for diagnosis of ovarian cancer: meta-analysis. Ultrasound Obstet Gynecol.

[27] Kaijser J, Sayasneh A, Van Hoorde K (2014). Presurgical diagnosis of adnexal tumours using mathematical models and scoring systems: a systematic review and meta-analysis. Hum Reprod Updat.

[28] Van Calster B, Timmerman D, Valentin L (2012). Triaging women with ovarian masses for surgery: observational diagnostic study to compare RCOG guidelines with an International Ovarian Tumour Analysis (IOTA) group protocol. BJOG.

[29] Anthoulakis C, Nikoloudis N (2014). Pelvic MRI as the “gold standard” in the subsequent evaluation of US-indeterminate adnexal lesions: a systematic review. Gynecol Oncol.

[30] Thomassin-Naggara I, Touissaint I, Perrot N, Rouzier R (2011). Characterization of complex adnexal masses: value of adding diffusion and perfusion MRI to conventional MR imaging. Radiology.

[31] Mohaghegh P, Rockall AG (2012). Imaging strategy for early ovarian cancer: characterization of adnexal masses with conventional and advanced imaging techniques. Radiographics.

[32] • Nougaret S, Tirumani SH, Addley H, Pandey H, Sala E, Reinhold C. Pearls and pitfalls in MRI of gynecologic malignancy with diffusion-weighted technique. Am J Roentgenol. 2013; 200:261–76*. Review of DWI MR imaging technique and illustration of typical pathologies as well as limitation of this technique.* 10.2214/AJR.12.9713 23345345

[33] Rockall A (2014). Diffusion weighted MRI in ovarian cancer. Curr Opin Oncol.

[34] Oh JW, Rha SE, Ohh SN, Park MY, Byun JY, Lee A (2015). Diffusion-weighted MRI of epithelial ovarian cancers: correlation of apparent diffusion coefficient values with histologic grade and surgical stage. Eur J Radiol.

[35] Kierans AS, Benenett GL, Mussi T (2013). Characterization of malignancy of adnexal lesions using ADC entropy: comparison with mean ADC and qualitative DWI assessment. J Magn Reson Imaging.

[36] Bernardin L, Dilks P, Liyanage S, Miquel ME, Sahdev A, Rockall A (2012). Effectiveness of semi-quantitative multiphase dynamic contrast-enhanced MRI as a predictor of malignancy in complex adnexal masses: radiological and pathological correlation. Eur Radiol.

[37] Thomassin-Naggara I, Bazot M, Daraï E (2008). Epithelial ovarian tumours: value of dynamic contrast-enhanced MR imaging and correlation with tumour angiogenesis. Radiology.

[38] Thomassin-Naggara I, Daraï E, Cuenod CA (2008). Dynamic contrast-enhanced magnetic resonance imaging: a useful tool for characterizing ovarian epithelial tumors. J Magn Reson Imaging.

[39] Thomassin-Naggara I, Balvay D, Aubert E (2011). Quantitative dynamic contrast-enhanced MR imaging analysis of complex adnexal masses: a preliminary study. Eur Radiol.

[40] • Thomassin-Naggara I, Aubert E, Rockall A. Adnexal masses: development and preliminary validation of an MR imaging scoring system. Radiology 2013; 267:432–43. *Retrospective assessment of feasibility of characterization of adnexal masses using the MRI scoring system by 2 blinded readers. The ADNEX MRI score was reproducible and a score of 4 or higher is highly associated with malignancy.* 10.1148/radiol.13121161 23468574

[41] Lee SI, Catalano OA (2015). Dehdashti. Evaluation of gynecologic cancer with MR imaging, ^18^ F-FDG PET/CT, and PET/MR imaging. J Nucl Med.

[42] Iver VR, Lee SI (2010). MRI, CT and PET/CT for ovarian cancer detection and adnexal characterization. AJR Am J Roentgenol.

[43] • Kandukuri SR, Rao J. FIGO 2013 staging system for ovarian cancer: what is new in comparison to the 1988 staging system. Curr Opin Obstet Gynecol 2015; 27:48–52. *This paper provides an excellent overview of the recent FIGO staging classification. It reviews the rationale for modification and compares it to the previous staging classification.* 10.1097/GCO.0000000000000135 25490382

[44] Prat J (2012). Ovarian carcinomas: five distinct diseases with different origins, genetic alterations, and clinico pathological features. Virchows Arch.

[45] Suh DH, Kim TH, Kim JW, Kim SY (2013). Improvements to the FIGO staging for ovarian cancer: reconsideration of lymphatic spread and intraoperative tumor rupture. J Gynecol Oncol.

[46] Berek JS (2009). Lymph node-positive stage IIIC ovarian cancer: a separate entity?. Int J Gynecol Cancer.

[47] Forstner R, Sala E, Kinkel K, Spencer JA (2010). ESUR guidelines: ovarian cancer staging and follow-up. Eur Radiol.

[48] Nougaret S, Addley HC, Colombo PE, Fujii S (2012). Ovarian carcinomatosis: how the radiologist can help plan the surgical approach. Radiographics.

[49] Ma FH, Cai SQ, Qiang JW (2015). MRI for differentiating primary fallopian tube carcinoma from epithelial ovarian cancer. J Magn Reson Imaging.

[50] Farmakis S, Vejdani K, Muzafffar R, Parkar N, Osman MM. Detection of metastatic disease in cardiophrenic lymph nodes: FDG PET/CT versus contrast-enhanced CT and implications for staging and treatment of disease www.frontiersin.org (2013). Accessed 19 Jan 2016. 10.3389/fonc.2013.00260 PMC3787306 24102048

[51] Kim TH, Lim MC, Kim SI, Seo SS (2015). Preoperative prediction of cardiophrenic lymph node metastasis in advanced ovarian cancer using CT. Ann Surg Oncol.

[52] Holloway BJ, Gore ME, A´Hern RP, Parson C (1997). The significance of paracardiac lymph node enlargement in ovarian cancer. Clin Radiol.

[53] Mironov O, Ishill NM, Mironov S, Vargas HA (2011). Pleural effusion detected at CT prior to primary cytoreduction of stage III or IV ovarian carcinoma: effect on survival. Radiology.

[54] Chang SJ, Bristow RE, Chi DS, Cliby WA (2015). Role of aggressive surgical cytoreduction in advanced ovarian cancer. J Gynecol Oncol.

[55] Hoskins WJ, McGuire WP, Brady MF, Homesley HD (1994). The effect of diameter of largest residual disease on survival after primary cytoreductive surgery in patients with suboptimal residual epithelial ovarian carcinoma. Am J Obstet Gynecol.

[56] Chang SJ, Hodeib M, Chang J, Bristow RE (2013). Survival impact of complete cytoreduction to no gross residual disease for advanced –stage ovarian cancer: a meta-analysis. Gynecol Oncol.

[57] Gomez-Hildago NR, Martinez-Cannon BA, Nick AM, Lu KH (2015). Predictors of optimal cytoreduction in patients with newly diagnosed advanced-stage epithelial ovarian cancer: time to incorporate laparoscopic assessment into the standard of care. Gynecol Oncol.

[58] Kehoe S, Hook J, Nankivell M, Jyson G, Kitchener H (2015). Primary chemotherapy versus primary surgery for newly diagnosed advanced ovarian cancer (CHORUS): an open-label, randomised, controlled, non-inferiority trial. Lancet.

[59] Aletti GD, Santillan A, Eisenhauer EL, Hu J (2007). A new frontier of quality of care in gynecologic oncology surgery: multi-institutional assessment of short term-outcomes for ovarian cancer using a risk adjusted model. Gynecol Oncol.

[60] Suidan RS, Ramirez PT, Sarasohn DM, Teitcher JB (2014). A multicenter prospective trial evaluating the ability of preoperative computed tomography scan and serum CA-125 to predict suboptimal cytoreduction at primary debulking surgery for advanced ovarian, fallopian tube, and peritoneal cancer. Gynecol Oncol.

[61] Borley J, Wilhelm-Benartzi C, Williamson R, Bharwani N (2015). Radiological predictors of cytoreductive outcomes in patients with advanced ovarian cancer. BJOG.

[62] • Sala E, Rockall AG, Freeman SJ, et al. The added role of MR imaging in treatment stratification of patients with gynecologic malignancies: what the radiologists needs to know. Radiology 2013; 266:717–740. *Summarizes all the essentials in uterine and ovarian cancer.* 10.1148/radiol.12120315 23431227

[63] Nelson BE, Rosenfield AT, Schwartz PE (1993). Preoperative abdominopelvic computed tomographic prediction of optimal cytoreduction in epithelial ovarian carcinoma. J Clin Oncol.

[64] Bristow RE, Duska LR, Lambrou NC (2000). A model for predicting surgical outcome in patients with advanced ovarian carcinoma using computed tomography. Cancer.

[65] Low RN, Barone RM (2012). Combined diffusion-weighted and gadolinium-enhanced MRI can accurately predict the peritoneal cancer index preoperatively in patients being considered for cytoreductive surgical procedures. Ann Surg Oncol.

[66] Low RN, Barone RM, Lucero J (2015). Comparison of MRI and CT for predicting the peritoneal cancer index (PCI) preoperatively in patients being considered for cytoreductive surgical procedures. Ann Surg Oncol.

[67] • Michielsen K, Vergote I, Op de Beeck et al. Whole–body MRI with diffusion-weighted sequence for staging of patients with suspected ovarian cancer: a clinical feasibility study in comparison to CT and FDG-PET/CT. Eur Radiol. 2014;24:889–901. *Prospective assessment of whole body MRI using DWI for staging ovarian cancer. Comparison of DWI to PET/CT and CT showed higher detection of mesenteric and serosal implants for DWI than CT or PET/CT. For thoracic metastases MRI was comparable to PET/CT.* 10.1007/s00330-013-3083-8 24322510

[68] Schmidt S, Meuli RA, Achtari C, Prior JO (2015). Peritoneal carcinomatosis in primary ovarian cancer staging: comparison between MDCT MRI and 18F-FDGPET/CT. Clin Nucl Med..

